# Reactivation of resolved hepatitis B virus infection combined with nephrotic syndrome in a patient after allogeneic haematopoietic stem cell transplantation

**DOI:** 10.1186/s12879-019-3690-3

**Published:** 2019-01-16

**Authors:** Jing-Wen Zhang, Xiang-Zhong Zhang, Yan-Ling Sun, Bing Long, Xiao-Zhen Wang, Xu-Dong Li

**Affiliations:** Department of Hematology, The Third Affiliated Hospital, Sun Yat-sen University, 600 Tianhe Road, Guangzhou, China

**Keywords:** Hepatitis B virus, Reactivation, Nephrotic syndrome, Haematopoietic stem cell transplantation, Immunosuppression

## Abstract

**Background:**

After allogeneic haematopoietic stem cell transplantation (allo-HSCT), Hepatitis B virus reactivation (HBVr) can be observed in patients with previous resolved Hepatitis B virus (HBV) infections. Nephrotic syndrome (NS) is the main clinical manifestation of HBsAg-positive glomerulonephritis. However, the development of HBVr combined with NS after allo-HSCT is uncommon.

**Case presentation:**

We presented a case of a 47-year-old female with acute myelogenous leukemia who underwent HLA-identical sibling allo-HSCT and achieved leukemia free survival. She had pretransplant serological markers of a resolved HBV infection (HBsAg-negative, anti-HBc and anti-HBs positive). However, she developed HBVr combined with nephrotic syndrome (NS) 16 months after HSCT. Her histological renal lesion was mesangial proliferative glomerulonephritis. IgA+, IgM+, and C1q deposits but not HBV antigens (HBsAg and HBcAg) were identified in her renal biopsy material. Long-term entecavir and immunosuppression resulted in decrease of HBV virus replication, amelioration of proteinuria and stabilisation of renal function.

**Conclusions:**

Entecavir combined with immunosuppression has efficacy in the treatment of HBVr combined with NS after allo-HSCT, but long course of treatment is needed. Closely monitoring and antiviral prophylaxis might be necessary for allo-HSCT recipients to prevent reactivation of resolved HBV infection and its related complications.

## Background

Hematopoietic stem cell transplantation (HSCT) is broadly undertaken with the aim of a complete cure of hematopoietic malignancies. Hepatitis B virus reactivation (HBVr) after allogeneic haematopoietic stem cell transplantation (allo-HSCT) is well known in HBsAg-positive carriers but has only occasionally been reported in patients with resolved Hepatitis B virus (HBV) infection [1, 2]. Resolved HBV infection is defined as follows: Previous known history of acute or chronic hepatitis B or the presence of anti-HBc with/without anti-HBs; HBsAg negative; undetectable serum HBV DNA; normal aminoleucine transferase (ALT) levels [3]. Recovery from an acute infection is usually assumed to provide lifelong protection against a future exposure to HBV. However, in individuals with resolved infection, HBV has been shown to persist in a ‘latent’ state in the liver and in peripheral blood mononuclear cells for years and possibly even lifelong [4]. With severe immunosuppression, HBsAg may reappear (reverse seroconversion) or viral replication may be detectable.

Renal dysfunction after HSCT is common. But the development of nephrotic syndrome (NS) after HSCT is uncommon and the incidence rate was reported to be 0.37–1.03% in adults [5]. Renal injury may also occasionally occur in patients with HBV infection and the incidence rate was about 3% [6]. The authors describe here a rare case with evidence of resolved HBV infection developed HBVr combined with NS after allo-HSCT.

## Case presentation

A 47-year-old female came to our hospital with a 20-day history of gingival bleeding and skin ecchymosis in September, 2014. Complete blood count showed leukocyte count 2,187,000/mm^3^, hemoglobin 8.6 g/dL, and platelet count 54,000/mm^3^. A bone marrow aspiration revealed 30% myeloblast. Chromosome analysis revealed 11q23 abnormality. An MLL-AF6 fusion transcript was detected by a reverse transcriptase polymerase chain reaction (RT-PCR). A diagnosis of acute myelogenous leukemia with 11q23/MLL translocations (high risk) was made. She entered remission after induction chemotherapy with idarubicin and cytarabine. She subsequently received two courses consolidation therapy with high dose cytarabine and one course of consolidation therapy with mitoxantrone and cytarabine. Then she underwent HLA-identical sibling HSCT in January, 2015. Conditioning include cytarabing, busulfan, semustine and cyclophosphamide. Graft-versus-host (GVHD) prophylaxis consisted of cyclosporine, methotrexate, and mycophenolate mofetil. Immunosuppression was tapered and then discontinued on September 17, 2015. She achieved trilineage engraftment and leukemia-free survival.

From onset to pretransplantation, monthly monitoring of HBV-related serum markersall showed that anti-HBs and anti-HBc were positive, HBsAg was negative, serum HBV DNA was undetectable, alanine aminotransferase (ALT) and aspartate aminotransferase (AST) were normal (below 40 IU/L). Her donor serology showed no previous HBV infection (HbsAg, anti-HBc and anti-HBs negative). The patient received entecavir at a dose of 0.5 mg once daily to prevent HBV reactivation and discontinued simultaneously with the cessation of immunosuppression in September 2015. In December 2015, laboratory tests showed serum transaminase levels were increased (AST 146 IU/L, ALT 163 IU/L), serum anti-HBs and anti-HBc turned negative, HBsAg was still negative and the HBV DNA titer was undetectable, and her renal function was normal. Liver chronic GVHD was considered and methylprednisolone was administered(8 mg/d for 10 days then 4 mg/d for a month). Then her serum transaminase levels returned to normal. She was then discharged from the hospital and told to monitor HBV-related serum markers and liver function monthly. But she did not have follow-up examinations in our out-patient clinic.

**Fig. 1:**
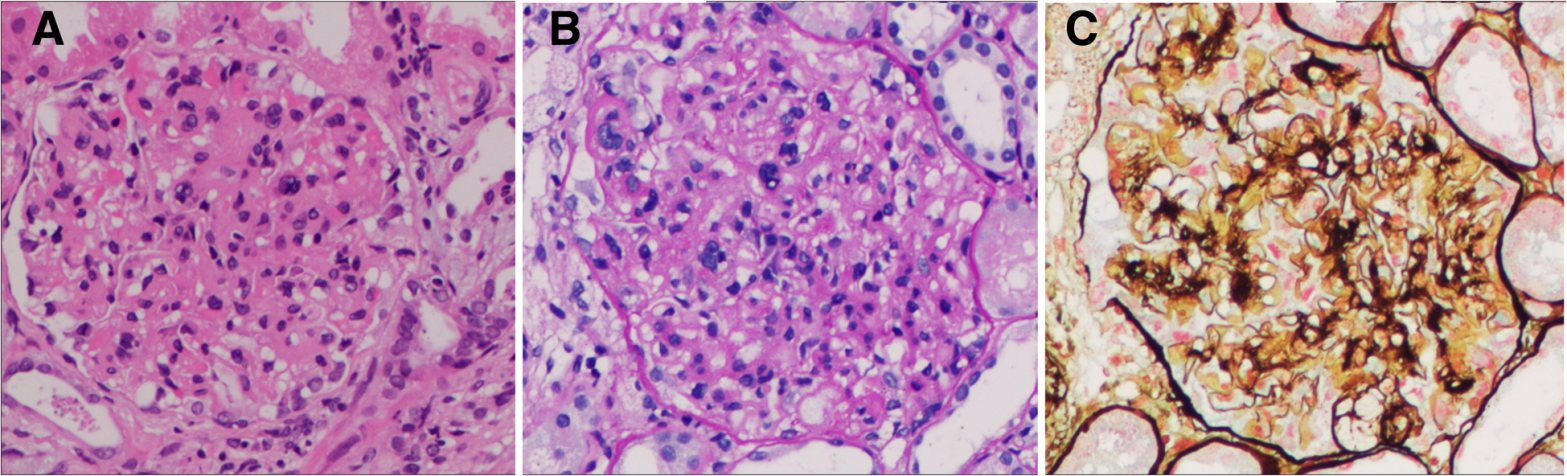
Image of renal biospy. The mesangial areas were slightly to moderately widened, with glomerular mesangial cells proliferation and extracellular matrix accumulation. (**a** HE staining, **b** PAS staining, **c** silver staining, 400x)

In May 2016, she presented to our hospital with moderate edema of both lower limbs and foamy urine for half a month. Her renal function was impaired (creatinine, 112umol/L), and NS was confirmed by low serum albumin (19.0 g/L), high cholesterol (10.21 mmol/L), and proteinuria (11.3 g/day). Detection of HBV-related serum markers showed that HBsAg and HBeAg turned positive, and the quantity of HBV DNAwas 1.7 × 10^8^ IU/ml. ALT and AST were still normal. Her liver and kidneys appeared normal on ultrasound examination. Two kidney biopsies containing 34 glomeruli were analysed morphometrically. Light microscopy revealed 4 glomeruli (11.7%) were completely collapsed; the rest mesangial areas were slightly to moderately widened, with glomerular mesangial cells proliferation and extracellular matrix accumulation; chronic tubulointerstitial damages existed, including focal tubular atrophy, tubular epithelial brush-border loss, focal interstitial fibrosis and monocytes infiltration; the capillary loops were open and there was no thickening of the glomerular basement membrane(Fig. 1). Immunofluorescence revealed deposits in segmental glomerular capillary loops and mesangial area; sample fluorescence distribution: IgA+; IgM+; C1q+, IgG-, C3-, HBsAg-, HBcAg-. Kidney pathology was consistent with mesangial proliferative glomerulonephritis.

She was immediately administered entecavirat a dose of 0.5 mg once daily to repress the replication of HBV DNA and valsartan at a dose of 800 mg once daily to reduce urinary protein. Two months later, the level of HBV DNA decreased to 2.4 × 10^4^ IU/ml. However, her renal function was still aggravated. The level of serum albumin was 16.5 g/L and creatinine was 125 umol/L. Then immunosuppressive therapy was started to treat kidney disease. Mycophenolate mofetil at a dose of 500 mg/12 h and prednisone at a dose of 20 mg/d were initiated and then gradually reduced. Her renal function was gradually improved. In April 2017, her serum albumin was 42.2 g/L and serum creatinine was 84 umol/L. But her urine albumin creatinine ratio was 844.13 mg/g and the quantity of HBV DNA was 9.5 × 10^2^ copies IU/ml, which meant she still had clinical albuminuria and persistent HBV replication. In July 2018, she was given mycophenolate mofetil at a dose of 500 mg/d and prednisone at a dose of 8 mg/d, and her urine albumin creatinine ratio declined to 48.57 mg/g. She continued to be treated with immunosuppressive therapy and antiviral therapy.

## Discussion and conclusions

According to previous reports, NS develops on average approximately 1 to 2 years after allo-HSCT [5, 7]. Its etiology may be related to drug toxicity, infection, or graft versus host disease (GVHD) [8]. In our case, the patient developed NS 16 months after transplantation and 8 months after the cessation of immunosuppression, so the etiology could not be drug toxicity.

NS has been reported to be a manifestation of chronic GVHD (cGVHD) [9, 10]. However, the incidence of NS associated with cGVHD is rare. GVHD as the definitive cause of the nephropathy should be interpreted with caution. GVHD-related NSwas considered present according to the following points: the development of NS immediately after the discontinuation or a dosage decrease of immunosuppression therapy, lymphocyte infiltration observed in the kidney, and the existence of other manifestations of cGVHD [10, 11]. However, in our case, NS was developed 6 months after the cessation of immunosuppression therapy; the patient had no manifestations of cGVHD in other organs; and lymphocytes infiltration was not observed in the kidneys. Therefore, there was no enough evidence to prove that the kidneys were the target of cGVHD.

This patient had a history of resolved HBV infection (HBsAg-negative, anti-HBc-positive and anti-HBs-positive) before HSCT. But after HSCT or immunosuppression, HBV reactivation can also be observed in patients with previously resolved HBV infections [1, 2]. Our patient received entecavir to prevent HBV reactivation, and discontinued simultaneously with the cessation of immunosuppression. Two months after entecavir withdrawal, she wasclinically suspected of having liver cGVHD based on her isolated AST/ALT elevation. She was then given a 40-day course of methylprednisolone (8 mg/d for 10 days then 4 mg/d for a month) without antiviral prophylaxis. Her serum transaminase levels returned to normal. However, 6 months later, she had a high HBV DNA viral load and developed NS. The relationship between HBV infection and kidney damage is complex. HBV associated glomerulonephritis is a major extra-hepatic organ disease after HBV infection, however, not all patients with HBV infection have kidney damage associated with HBV but might exhibit HBV infection with primary glomerulonephritis [6]. The current diagnostic criteria for HBV associated glomerulonephritis include: 1) the presence of a serum HBV antigen; 2) the diagnosis of glomerulonephritis with the exclusion of other types of secondary nephritis; and 3) the presence of renal HBV antigen, which is required for the diagnosis of HBV associated glomerulonephritis [6, 12, 13]. In our case, we were unable to find evidence of HBV antigens in her kidney biopsy samples to prove conclusively that kidney damage was caused by HBV infection. Her renal biopsy also showed IgA, IgM and C1q positive immunoflurescence staining in kidney tissues could indicate immunological abnormality was related to the development of NS.

HBVr combined with NS after allo-HSCT is a rare clinical problem. Its pathogenesis is not completely clear, so no clear treatment principles are currently available. In this case, we could notclarify the exact cause of NS, as the kidney biopsy findings are not specific for either HBV associated glomerulonephritis orcGVHD-related NS. Nevertheless, antiviral therapy combined withimmunosuppressive therapy gradually improved the outcome. Firstly our patient was administered entecavir to repress the replication of HBV DNA. Two months later, serum HBV DNA levels decreased more than 3 logs/ml but her renal function continued to deteriorate, which was consistent with there were no HBV antigens in her kidney biopsy samples. Since the kidney biopsy suggested immunological abnormality was related to the development of NS, immunosuppressants were then given to inhibit immune and inflammatory responses. After 2 years of antiviral and immunosuppressive therapy, she achieved complete remission of NS, but she still had microalbuminuria.

This case revealed thatreactivation of resolved HBV infectionshould be paid more attention after HSCT. There has been sporadic reports of HBVr in HSCT recipients with antibodies against HBs and HBc antigens, but the actuarial risk might be high [1, 2]. Knoll et al. [14] monitored seven patients with pretransplant anti-HBs and anti-HBc antibodies and found that reverse seroconversion (from anti-HBs to HBsAg) was observed in six recipients. A previous study suggested that the absence of anti-HBs is a predictor of HBVr in patients with resolved hepatitis B receiving chemotherapy [15]. So, when our HSCT recipient’s serum anti-HBs turned negative, we should have realized she was at high risk of HBVr and taken precautions to prevent it from happening. The current approach to preventing reactivation of resolved HBV infection is controversial. According to the guidelines of Asian-Pacific clinical practice guidelines on the management of hepatitis B, HBsAg negative and anti-HBc positive subjects should be closely monitored by HBV DNA during and at least 12 months after immunosuppressive therapy and antiviral treatment should be started once the HBV DNA is detectable [16]. However, according to the guidelines of European association for the study of the liver, anti-HBc positive subjects undergoing stem cell transplantation, antiviral prophylaxis is recommended, and prophylaxis should continue for at least 18 months after stopping immunosuppression and monitoring should continue for at least 12 months after prophylaxis withdrawal [17]. The above guidelines both emphasize the importance of close monitoring. However, our patient did not undergo follow-up surveillance testing after she completed 40-day course of methylprednisolone, which revealed our deficiency in patient’s education. Although HBV-associated liver disease after allo-HSCT is often temporary and not serious, fatal liver damage has been reported in some cases [18–20]. In our case, entecavir combined with immunosuppressive therapy could gradually improve the outcome of NS combined with HBVr. However, long-term immunosuppressive therapy can activate HBV replication and increase the risk of leukemia relapse; long-term antiviral treatment can lead to the emergence of drug-resistant mutant viruses. Therefore, closely monitoring and antiviral prophylaxis might both be necessary for HBsAg negative and anti-HBc positive patients undergoing HSCT.

In conclusion, entecavir combined with immunosuppression has efficacy in the treatment of HBVr combined with NS after allo-HSCT, but long course of treatment is needed. Closely monitoring and antiviral prophylaxis might be necessary for allo-HSCT recipients to prevent reactivation of resolved HBV infection and its related complications.

## Abbreviations

allo-HSCT: allogeneic haematopoietic stem cell transplantation
ALT: aminoleucine transferase
AST: aspartate aminotransferase
cGVHD: chronic graft versus host disease
GVHD: graft versus host disease
HBV: hepatitis B virus
HBVr: hepatitis B virus reactivation
HSCT: hematopoietic stem cell transplantation
NS: nephrotic syndrome
